# Dandy-Walker Malformation With Hydrocephalus: Diagnosis and Its Treatment

**DOI:** 10.7759/cureus.25287

**Published:** 2022-05-24

**Authors:** Swaragandha S Jadhav, Avinash Dhok, Kajal Mitra, Sharjeel Khan, Sandeep Khandaitkar

**Affiliations:** 1 Radiology, N. K. P. Salve Institute Of Medical Sciences & Research Centre, Nagpur, IND; 2 Radiodiagnosis and Imaging, N. K. P. Salve Institute Of Medical Sciences & Research Centre, Nagpur, IND; 3 Forensic Medicine, N. K. P. Salve Institute Of Medical Sciences & Research Centre, Nagpur, IND; 4 Oral and Maxillofacial Surgery, Vidya Shikshan Prasarak Mandal (VSPM) Dental College & Research Centre, Nagpur, IND

**Keywords:** cisterna magna, mri brain, dandy-walker, ventriculoperitoneal shunting, hydrocephalus

## Abstract

Dandy-Walker malformation is an uncommon type of brain malformation that occurs during embryonic development of the cerebellum and fourth ventricle. A case of Dandy-Walker malformation with hydrocephalus is being elaborated. The patient was operated on by the neurosurgery department and underwent ventriculoperitoneal shunting. The patient was stable and improved during the postoperative period.

## Introduction

Dandy-Walker deformity is an uncommon posterior fossa anomaly marked by vermis agenesis or hypoplasia, as well as cystic expansion of the fourth ventricle [1]. During the embryonic development of the cerebellum and fourth ventricle, this causes the tentorium to be displaced upwards and the posterior fossa gets enlarged. The association of hydrocephalus with Dandy-Walker malformation is due to blockage of normal cerebrospinal flow leading to an excessive amount of fluid accumulating in and around the brain and causing an increase in intracranial pressure and head circumference, which ultimately causes neurological impairment [1]. The majority of individuals had hydrocephalus at the time of diagnosis. Many people go years without seeing any symptoms and the rest are diagnosed earlier due to comorbidities. Hydrocephalus and posterior fossa symptoms are routinely treated with surgical methods such as ventriculoperitoneal and cystoperitoneal shunting.

## Case presentation

A non-consanguineous marriage-born eight-year-old male came with complaints of throbbing headache, three episodes of seizures in eight days, and vomiting for two days. A history of poor scholastic performance was present. No history of fever, difficulty in vision, ataxia/movement incoordination, and hearing loss was present. On general examination, the patient was of short stature. He was conscious, and well oriented to time, place, and person. His head circumference was increased as per his age. The ophthalmic and audiometric examinations were normal. The patient was able to count objects, say his full name, ride a tricycle, and draw a circle. As per the clinical examination, he has achieved milestones up to the age of three years. The patient was vitally stable. The systemic examination was within normal limits. Immunisation was up to date. The patient’s mother never had ultrasound scans during her antenatal care (ANC) period. She did not give a history of fever during pregnancy, or a history of miscarriage. It was a normal full-term vaginal delivery with a baby weight of 3 kg. He immediately cried after birth. The patient was advised magnetic resonance imaging (MRI) of the brain for further evaluation.

**Figure 1:**
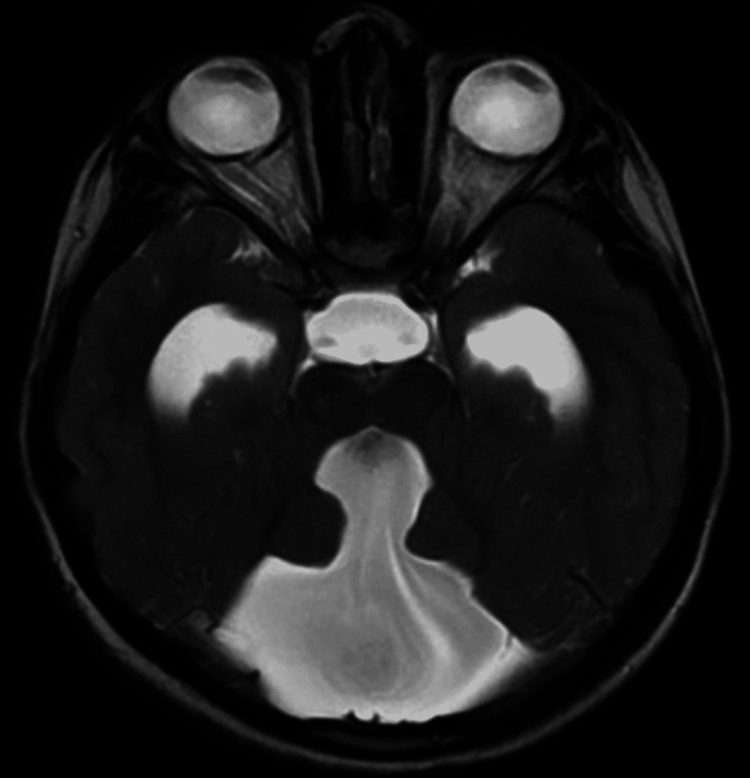
Brain MRI plain axial T2-weighted image shows dilated fourth ventricle communicating with a large posterior fossa cyst.

**Figure 2:**
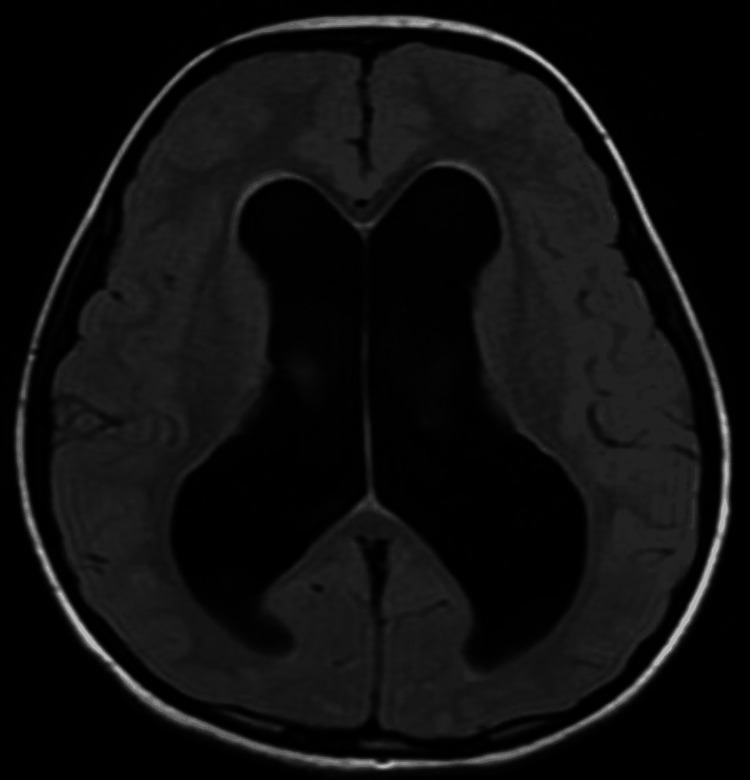
Brain MRI plain axial fluid-attenuated inversion recovery (FLAIR) image shows dilated bilateral lateral ventricles with generalised cerebral oedema.

**Figure 3:**
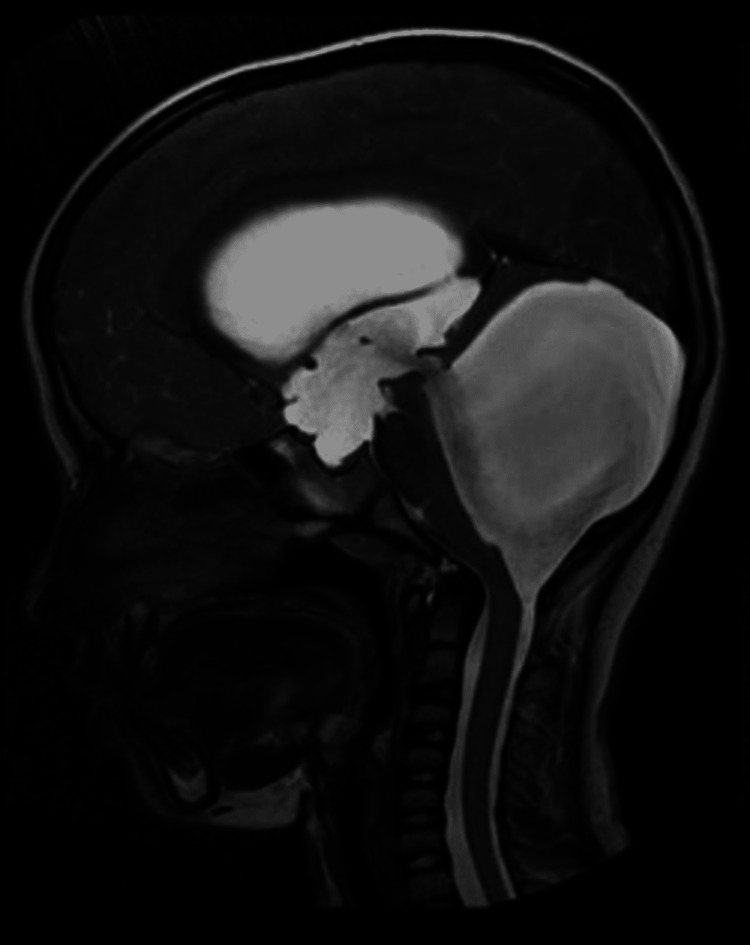
Brain MRI plain sagittal T2-weighted image shows dilated ventricular system with the fourth ventricle communicating with a large posterior fossa cyst compressing the brain stem anteriorly and pushing the hypoplastic cerebellar hemisphere superiorly.

There was dilatation of the fourth ventricle communicating with a large posterior fossa cyst noted on axial T2-weighted imaging (T2WI), axial fluid-attenuated inversion recovery (FLAIR), and sagittal T2WI sequences on MRI of the brain (Figures 1-3).

On all the above-mentioned sequences, generalised cerebral oedema was noted with absent vermis and cerebellar hypoplasia (Figures 1, 2). The dilatation of the entire ventricular system with the fourth ventricle was noted communicating with a large posterior fossa cyst compressing the brain stem anteriorly and pushing the hypoplastic cerebellar hemisphere superiorly. There was marked dilatation of bilateral lateral ventricles and third ventricles with ballooning of the supraoptic and infundibular recess noted. Funnelling of the superior part of the aqueduct was seen. The cervicomedullary junction appeared narrow likely due to compression by the large posterior fossa cyst. Sella appeared J shaped with compressed and inferiorly displaced pituitary gland due to enlarged infundibular recess. The corpus callosum was compressed because of dilated ventricles (Figure 3). Minimal periventricular oozing was noted. Cella media index was 2.5, indicating hydrocephalus. Based on the above imaging features, a diagnosis of Dandy-Walker malformation with moderate hydrocephalus was made. The patient underwent ventriculoperitoneal shunting four days later in the neurosurgery department. The patient was stable and improved during the postoperative period. The postoperative visits were inconclusive.

## Discussion

Dandy-Walker malformation is an extremely rare type of brain abnormality. Its incidence is estimated to be one in 25,000-30,000. As compared to males, females are more likely to be affected. It is caused by abnormalities in the early embryonic development of the cerebellum and its surrounding structures [1]. Most of the time, patients have a non-specific clinical presentation. Complaints mainly depend upon the severity of hydrocephalus. Macrocephaly is the most prevalent symptom present in the early neonatal period.

Developmental delay, flaccidity or spasticity, poor coordination, ataxia, and occasionally increased head circumference with increased intracranial pressure due to hydrocephalus are all common symptoms of Dandy-Walker malformation. Seizures afflict 15-30% of people who are affected. Severe hydrocephalus can lead to respiratory failure. The age of diagnosing hydrocephalus varies based on the development and severity of the condition, as well as the existence of additional birth defects or medical issues.

When examining the fetal brain, ultrasound is usually the first imaging modality used. Ultrasound can assess head circumference, bilateral thalami, lateral ventricles, choroid plexus anatomy, cavum septum pellucidum, cerebellum, cisterna magna size, nuchal fold, and spine with good accuracy. Other CNS abnormalities that are typically associated with Dandy-Walker malformation may be visible on imaging. The cisterna magna is measured as part of the prenatal brain ultrasonography examination. During prenatal fetal ultrasound evaluation, a prominent cisterna magna may raise concerns about congenital posterior fossa anomalies [1]. After the 20th week of pregnancy, MRI of the brain is superior in diagnosing CNS anomalies as compared to ultrasonography [2].

Mega cisterna magna is the differential diagnosis of Dandy-Walker malformation and is characterised by a larger posterior fossa despite the normal cerebellar size. An expanded fluid collection beneath and typically behind the cerebellum is linked to the increased size. Although developmental problems are less severe than those seen in Dandy-Walker malformation or cerebellar vermis hypoplasia.

Cerebellar vermis hypoplasia is characterised by a tiny vermis without the conspicuous upward rotation, and cystic expansion of the fourth ventricle or expanded posterior fossa is seen in typical Dandy-Walker malformation. This deformity is also known as the "Dandy-Walker variant," which might be misleading. After the cerebellar vermis has fully grown in the 18th week of pregnancy, ultrasonography can be used to provide a prenatal diagnosis. To confirm the diagnosis, MRI can be done [3]. A definitive diagnosis is made by karyotype and postnatal imaging [4].

The majority of patients have clinical features of elevated intracranial pressure, which is usually caused by hydrocephalus or a posterior fossa cyst. As a result, most treatments try to reduce intracranial pressure, usually through surgery.

Ventriculoperitoneal or cystoperitoneal shunts are two surgical options. Endoscopic procedures, such as endoscopic third ventriculostomy, may be appropriate for other patients [5]. In 2009, Agrawal and Thakur published a case report on a three-year-old full-term male infant with an abnormal head circumference and respiratory distress [6]. CT of the brain revealed that the fourth ventricle was dilated and the cerebellar hemispheres were hypoplastic with splaying and agenesis of the corpus callosum in a patient with Dandy-Walker deformity. These conclusions held true in our situation. The findings of vermian hypoplasia, a posterior fossa cyst communicating with the ventricle, supratentorial hydrocephalus, and bilateral subdural collections were described by Mallikarjun et al. in a case of a 10-year-old girl diagnosed with Dandy-Walker malformation on CT of the brain in 2010 [7]. To prevent bacterial endocarditis, all dental treatments were administered with antibiotic prophylaxis, especially for the youngster who had a ventriculoatrial shunt.

## Conclusions

Dandy-Walker malformation is an uncommon type of brain malformation in which the vermis agenesis or hypoplasia results in ventricular dilatation and hydrocephalus. Precise antenatal diagnosis may be achievable, but if missed, it can be diagnosed postnatally on CT or plain MRI of the brain. To alleviate the patient's symptoms, ventriculoperitoneal and cystoperitoneal shunting is the most effective treatment and can prevent further neurological impairment. This case highlights the significance of the early diagnosis of Dandy-Walker malformation and timely surgical intervention for reducing intracranial pressure.

## References

[1] Zamora EA, Ahmad T (2022). Dandy Walker Malformation. https://www.ncbi.nlm.nih.gov/books/NBK538197/.

[2] Correa GG, Amaral LF, Vedolin LM (2011). Neuroimaging of Dandy-Walker malformation: new concepts. Top Magn Reson Imaging.

[3] Kline-Fath BM, Calvo-Garcia MA (2011). Prenatal imaging of congenital malformations of the brain. Semin Ultrasound CT MR.

[4] Nyberg DA, Cyr DR, Mack LA, Fitzsimmons J, Hickok D, Mahony BS (1988). The Dandy-Walker malformation prenatal sonographic diagnosis and its clinical significance. J Ultrasound Med.

[5] Mohanty A, Biswas A, Satish S, Praharaj SS, Sastry KV (2006). Treatment options for Dandy-Walker malformation. J Neurosurg.

[6] Agrawal S, Thakur P (2009). Dandy-Walker malformation. BMJ Case Rep.

[7] Mallikarjun K, Vatsala V, Bhayya DP (2010). Dandy-Walker syndrome — a rare case report. J Adv Oral Res.

